# Notes on Two Cases of Compound Comminuted Fracture of the Skull

**Published:** 1881-03

**Authors:** S. H. Benton


### NOTES ON TWO CASES OF COMPOUND COMMINUTED FRACTURE OF THE SKULL.

BY DR. S. H. BENTON.

Willie W., aged 15 years, a clerk in a store in this city, on the evening of April 26th, 1879, was playing a game called "duck on the rock," and while in the act of stooping to place his duck on the rock was struck on the head by a stone weighing about eight pounds, thrown by the brother, a distance of some two rods, which caused a compound comminuted fracture of the lower and posterior part of the parietal and upper part of the temporal bones of the right side. I saw the patient about an hour after the accident. Found him unconscious and in convulsions, pupils dilated, pulse 30 per minute. Drs. Kitchey and Davis were called in consultation. The case seemed hopeless, but trephining was decided upon and done at once.

By enlarging the wound in the soft parts, we found a very large amount of bone depressed, and at least two tablespoonfuls of brain tissue exuded. We could not introduce the elevator, and a circular section of bone was thereupon removed, which enabled us to take out the fragments, measuring about 2x3 inches. The membranes were extensively lacerated, and we encountered considerable hæmorrhage. Convulsions ceased at once, but complete return of consciousness did not occur for three or four days. The pulse remained at 50 for about two days, after which it gradually regained its normal frequency. The wound was left open, and carbolized dressings used, and in about three weeks it healed by granulation.

Case II. Nov. 13, 1879, was called for to see John D; aged 7 years; residing with his parents at McClintockville. Found that the lad, while trying to catch a horse, was kicked on the head and carried to the house unconscious, in which condition I found him. I discovered a compound comminuted fracture of the frontal bone over the left orbit. Decided to trephine at once, and with the assistance of Dr. Miller, three fragments, in all about 2x2 inches, were removed. As in the former case, a portion of the brain matter was lost at the time of operating. The child became conscious in a half hour and remained so. The pulse did not exceed 60 per minute for a week. On the third day after the accident, a "hernia cerebri," about as large as a walnut, was removed from the wound with the knife. There was no special medication used in this case, cleanliness of the wound being the essential feature in its treatment, and with this it closed readily.

The two points of interest in both instances appear to be the extensive laceration of the membranes of the brain, without the production of meningitis, and the loss of brain substance, without corresponding impairment of the intellectual faculties. The cases suggest other topics for consideration, but these two points seemed to be made prominent even by the simple outline here given.

